# Differences in Root Nitrogen Uptake Between Tropical Lowland Rainforests and Oil Palm Plantations

**DOI:** 10.3389/fpls.2020.00092

**Published:** 2020-02-25

**Authors:** Nur Edy, Upik Yelianti, Bambang Irawan, Andrea Polle, Rodica Pena

**Affiliations:** ^1^ Forest Botany and Tree Physiology, University of Goettingen, Göttingen, Germany; ^2^ Department of Agrotechnology, Tadulako University, Palu, Indonesia; ^3^ Department of Biology, University of Jambi, Jambi, Indonesia; ^4^ Department of Forestry, University of Jambi, Jambi, Indonesia

**Keywords:** N stable isotope, N status, N uptake efficiency, tropical perennial crops, oil palm, rainforest, loam Acrisol

## Abstract

Conversion of lowland tropical rainforests to intensely fertilized agricultural land-use systems such as oil palm (*Elaeis guineensis*) plantations leads to changes in nitrogen (N) cycling. Although soil microbial-driven N dynamics has been largely studied, the role of the plant as a major component in N uptake has rarely been considered. We address this gap by comparing the root N contents and uptake in lowland rainforests with that in oil palm plantations on Sumatra, Indonesia. To this aim, we applied ^15^N-labeled ammonium to intact soil, measured the ^15^N recovery in soil and roots, and calculated the root relative N uptake efficiency for 10 days after label application. We found that root N contents were by one third higher in the rainforest than oil palm plantations. However, ^15^N uptake efficiency was similar in the two systems. This finding suggests that lower N contents in oil palm roots were likely caused by plant internal utilization of the absorbed N (e.g., N export to fruit bunches) than by lower ability to take up N from the soil. ^15^N recovery in roots was primarily driven by the amount of root biomass, which was higher in oil palm plantation than rainforest. The oil palms unveiled a high capacity to acquire N, offering the possibility of enhancing sustainable plantation management by reducing N fertilizer application.

## Introduction

Conversion of tropical rainforests to cash crop monocultures is globally increasing (Curran et al., 2004; Corley, 2009; Hansen et al., 2009) triggering rural development and often improving the living standards of the smallholder farmers (Euler et al., 2017). The economic benefits have, however, ecological trade-offs such as negative consequences for biogeochemical cycles of carbon and nitrogen (N) and eventually Earth's climate (Drescher et al., 2016; Guillaume et al., 2018).

Tropical soils generally have large pools of available N with high N-cycling rates (Hedin et al., 2009). Rainforest conversion to crop monocultures involves clearing, draining, and burning and thereby, decreases soil fertility (Dechert et al., 2004; Bringhurst and Jordan, 2015; Guillaume et al., 2016). Soil N dynamics, which determine the amount of plant-available N, may change under the rainforest transformation. For example, in Sulawesi, conversion of the lower montane forests to cornfields resulted in the slowdown of N cycling by decreasing the gross N mineralization and turnover rates of ammonium (NH_4_^+^) and microbial N pools (Corre et al., 2006).

To alleviate the soil N-supplying capacity for high crop yields, the addition of N fertilizers is common practice in the tropical cash crop systems (Allen et al., 2015; Kotowska et al., 2015). One of the most productive and widespread tropical rainforest conversion systems is oil palm plantations (FAO, 2018). Oil palm fertilization involves both economic (46 to 85% of the total plantation costs, Pardon et al., 2016) and ecological costs (enhanced N leaching and nitrous oxide emissions to the atmosphere, Dislich et al., 2017; Hassler et al., 2017; Hewitt et al., 2009; Veldkamp et al., 2008). For a reduction of the high N fertilization costs, a comprehensive understanding of soil N cycling in this conversion system is required.

Soil N processes hinge on plant and soil microbial components, and the competitive interactions between them (Groenigen et al., 2015). Tropical rainforest conversion to oil palm plantations alters both soil microbial communities (Sahner et al., 2015; Schneider et al., 2015; Berkelmann et al., 2018; Brinkmann et al., 2019) and plant diversity (Rembold et al., 2017). Although soil N transformation processes and microbial dynamics (Allen et al., 2015), as well as N loss pathways (Dislich et al., 2017; Hassler et al., 2017) have been studied in this conversion system, the ability of plants to take up N has remained unknown. Plant N acquisition has been investigated either in tropical rainforests (Houlton et al., 2006; Hedin et al., 2009) or in oil palm plantations (Pardon et al., 2016 and references therein), but comparative studies of plant N uptake under similar climatic conditions and soil types in the rainforest-oil palm conversion system are missing.

Jambi region on Sumatra (Indonesia) represents, besides other Southeast Asian regions, a hotspot of conversion of tropical lowland rainforests into oil palm plantations (Miettinen et al., 2011; Margono et al., 2014; Dislich et al., 2017). Oil palm is a highly productive (Barcelos et al., 2015) and resource-demanding species (Pardon et al., 2016). N inputs through mineral fertilization differ between the smallholders and the large agro-industrial plantations, but in both cases, they are commonly excessive (Woittiez et al., 2018). In the smallholder plantations in Jambi, the average is 30 kg N ha^−1^ year^−1^ with a massive variation of ± 150% between plantations (Moulin et al., 2017). Woittiez et al. (2018) reported an average of 166 kg N ha^−1^ year^−1^ in the smallholder plantations and Darras et al. (2019) reported 260 kg N ha^−1^ year^−1^ in a large plantation while the demand of a plantation producing 20 tons fresh fruit bunches per year has been reported as 129 kg N ha^−1^ year^−1^ (Ng et al., 1999). Supplementary, the return of empty fruit bunches to the plantations may bring 78 to 228 kg N ha^−1^ year^−1^ (Pardon et al., 2016). Less than 1% of the smallholders use organic fertilizers (Woittiez et al., 2018).

We conducted our study in loam acrisol soils (Harapan area on Sumatra, Indonesia), where the mineral soluble N pools amount about 4 mg N kg^−1^ soil (measured to a depth of 5 cm) with no differences between the oil palm plantations and lowland rainforests (Allen et al., 2015). Nearly 75% of the soluble N pool was present as NH_4_^+^ (Allen et al., 2015). The mineralization rates of about 4 to 5 mg N kg^−1^ soil day^−1^ and immobilization rates of about 2 to 3 mg N kg^−1^ soil day^−1^ also did not vary between forest and plantation soils (Allen et al., 2015). However, despite similar N availability, the roots from rainforests exhibited by about 50% higher N contents than oil palm roots (Sahner et al., 2015). So far, the reasons for these differences are unknown. Plant ability to take up N depends on differences in root morphological traits, such as the number of root tips and root tip vitality, and association with different microbial symbionts (Pena et al., 2013; Pena and Polle, 2014). The intensive management in the plantations negatively affects all these aspects (Sahner et al., 2015).

The goal of this study was to compare N uptake of mixed rainforest roots with roots in oil palm plantations. We hypothesized that root N contents are lower in oil palm than rainforest roots because rainforest roots have higher uptake efficiency and thus, acquire more N than oil palm roots. Alternatively, we hypothesized that higher root mass in oil palms plantations than in rainforests results in reduced N contents when both root system have similar N uptake efficiencies. To test these hypotheses, we applied ^15^N-labeled NH_4_^+^ to intact soil and traced the dynamics of the N-label in soil and roots. We measured how much from the initial N input was recovered in the soil and roots in lowland rainforests and oil palm plantations and estimated root uptake efficiency as the ratio of newly acquired N in roots per newly available N in the soil. All analyses were conducted in the same plots, where previously the soil characteristics, soil N transformation processes, and microbial dynamics (Allen et al., 2015), as well as root traits (Kotowska et al., 2015; Sahner et al., 2015), have been reported.

## Materials And Methods

### Study Site and Experimental Set-Up

This study was conducted in the Harapan landscape (Jambi Province, Sumatra, Indonesia). The mean annual temperature during the period 1991–2011 was 26.7 ± 0.2°C, and the mean annual precipitation was 2,235 ± 381 mm (Drescher et al., 2016). Integrated solar irradiance for the same period was 36.12 ± 0.28 (MJ m^−2^ day^−1^) (NASA Prediction of Worldwide Energy Resource (POWER); https://power.larc.nasa.gov/). The soil type is loam acrisol with a clay content of 26 ± 2 and 33 ± 4%, and pH values of 4.3 ± 0.0 and 4.5 ± 0.1 in the rainforest and oil palm plantation plots, respectively (Allen et al., 2015). The oil palm plots were located in smallholder plantations with 12 to 19-year-old trees. The tree density was 658 ± 26 trees ha^−1^ in the rainforest, and 140 ± 4 trees ha^−1^ in the plantations (Rembold et al., 2017). The plots in the Harapan rainforest area are dominated by members of *Aporosa* spp., Burseraceae spp., Dipterocarpaceae spp., Fabaceae spp., Gironniera spp., Myrtaceae spp., Plaquium spp., *Porterandia* sp., and *Shorea* spp. (Rembold et al., 2017).

The oil palm plantations were fertilized two times per year, once in the rainy and once in the dry season. The most commonly used fertilizers were the compound NPK, potassium chloride, and urea. The fertilizer addition was 550 kg NPK-fertilizer ha^−1^ year^−1^ that resulted into 88 kg N ha^−1^ year^−1^, 38 kg P ha^−1^ year^−1^, and 73 kg K ha^−1^ year^−1^. The understory was controlled by herbicides (Gramoxone and Roundup), which were applied twice a year at an average rate of 2 to 5 L herbicide ha^−1^ year^−1^ (Allen et al., 2015). Both the nutrient application and weed control were conducted inside a circle with a 2 m radius around each palm base. The interrows were treated with herbicides and fertilizers once a year. Pruning took place twice a year, and the harvesting frequency was 10 days. The research plots were established in the frame of the collaborative research center “Ecological and Socio-economic Functions of Tropical Lowland Rainforest Transformation Systems” (EFForTS, http://www.uni-goettingen.de/crc990). The plots are localized in the rainforest (core plots, HF2, HF3, and HF4) and oil palm plantations (core plots, HO2, HO3, and HO4) at a distance of at least 1.5 km each from another (**Table 1**). Each plot was 50 m x 50 m and contained five 5 x 5 m subplots, from which three (always the a, b, c, situated at least 15 m apart each from another, see a plot layout in **Figure S1**) were used for this experiment. The subplots were at fixed positions, randomly assigned at the beginning of plot establishment. The plot design and plot history are described in Drescher et al. (2016).

**Table 1:** Plot location used for ^15^N soil labeling in the Harapan district on Sumatra (Indonesia).

Plot	Land-use type	Latitude	Longitude	Elevation (masl)
HF2	Rainforest	S 02°09'49.3”	E 103°20'03.8”	74 m
HF3	Rainforest	S 02°10'42.5”	E 103°19'58.9”	51 m
HF4	Rainforest	S 02°11'15.4”	E 103°20'34.7”	71 m
HO2	Oil palm plantation	S 01°53'00.4”	E 103°16'03.1”	62 m
HO3	Oil palm plantation	S 01°51'27.6”	E 103°18'28.2”	57 m
HO4	Oil palm plantation	S 01°47'12.8”	E 103°16'14.6”	48 m

The rainforest plots are located in a natural reservation and the oil palm plots in smallholder plantations.

### ^15^N Labeling

In each subplot, at equal distances, for a precise delimitation of stable isotope labeling application spots, five small (1 cm height) PVC tubes of 5 cm diameter were placed on the soil. The soil labeling occurred inside the palm circle in the oil palm plantations at about 1 m from the tree stem. Similarly, in the forest subplots, large trees were chosen, and the labeling was conducted about 1 m from the trunk. The understory was avoided in the forest and not present in the oil palm plantation. The labeling solution was added into the PVC tube delimited-area and dripped into the soil. The labeling solution consisted of 40 ml of 2.0 mM ^15^NH_4_Cl (99% ^15^N, CK Isotopes, Leicestershire, United Kingdom) corresponding to 1.2 mg ^15^N applied on a soil area of 0.096 m^2^. This amount represented 37% in the forest and 30% in the oil palm plantations from the soil NH_4_^+^ pool (calculations after Allen et al., 2015). To ensure sufficient enrichment by a minimal NH_4_^+^ pool alteration, we used 99-atom% ^15^N (Davidson et al., 1991; Di et al., 2000). The irrigation conditions were chosen after pre-tests with different volumes of Brilliant Blue FCF solution (15.0 g l^−1^, SERVA Electrophoresis GmbH, Heidelberg, Germany, according to Wu et al., 2011). The Brilliant Blue FCF solution was traceable to a depth of about 10 cm after 24 h by a very small horizontal spread.

### Collection of Soil and Roots and Biomass Determination

After label application, the soil was collected using a metal soil corer of 5 cm diameter and 10 cm depth. One of the five soil cores per subplot was extracted immediately before application of the labeling solution (0.0 h). The other soil cores were collected at 0.16 (4 h), 1.0, 3.0, and 10.0 days after label application. In total, we collected 180 samples (90 per rainforest, 90 per oil palm plantation plots), with 9 replicates per time point.

Within 4 h after collection, the samples were transported in cooled thermo boxes to the field laboratory. The soil cores were immediately weighed to register the fresh biomass. Subsequently, the roots were manually collected from the soil cores, rinsed with distilled water, separated into fine and coarse roots, and weighed. The roots with a diameter of less than 2 mm were regarded as fine roots (Vogt et al., 1983). In the oil palm, those roots belong to tertiary roots (RIII) of 0.5–1.5 mm diameter and quaternary (RIV) roots of 0.2–0.5 mm diameter (Purvis, 1956; Jourdan and Rey, 1997). Here, we defined roots of diameters > 2 mm as coarse roots.

Fine roots were inspected for ectomycorrhizal specific structures (i.e., root tip fungal mantle) or nitrogen-fixing nodules, under a compound microscope. None of those structures was found. All root tips were classified as living and dead and counted accordingly, as described by Pena et al. (2010) and Sahner et al. (2015).

Samples of soil, fine roots, and coarse roots were dried at 45°C for 5 days and weighed to register the dry biomass.

Root density was calculated based on the volume of the collected soil core (196 cm^3^) as:

$$\mathrm{Root density}\left(\mathrm{mg}\;cm^{-3}\right)=\left(\frac{total\;root\;dry\;biomass\;}{196\;}\right)$$

The soil relative water content (RWC) was calculated as:

$$RWC\;\left(\%\right)=\frac{\left(fresh\;soil\;weight-dry\;soil\;weight\right)}{fresh\;soil\;weight}\times 100$$

The root tip density was calculated as the number of root tips per unit volume; root tip vitality as the percentage of living root tips from the total number of root tips.

### Determination of Carbon, Total N, and ^15^N

Dry soil and root samples were ground to a fine powder in a ball mill (MM200, Retsch, Haan, Germany). Aliquots of soil, fine roots, and coarse roots were weighed in tin capsules with a size of 4.96 mm for root samples and 5.99 mm for soil (IVA Analysentechnik e.K., Meerbusch, Germany).

The amounts of ^15^N, total N, and C in fine root, coarse root, and soil samples were measured at the Kompetenzzentrum for Stable Isotopes (KOSI, Göttingen University), using an elemental analyzer (NA 1108, Fisons, Rodano, Italy) coupled to an isotope ratio mass spectrometer (Delta Plus, Thermo Finnigan MAT GmbH, Bremen, Germany). The working standards (glutamic acid), calibrated against the international standards USGS 41 (δ^15^N_air_ = 47.600) for ^15^N, were measured after each tenth sample.

The ratio of ^15^N/^14^N was calculated as:

N15(atom%)=[(δ15N1000)+1]×Rstandard∗100

where ^δ15^N refers to the difference in ^15^N enrichment between the sample and atmospheric N_2_ as standard (Rstandard =0.0036765).

The abundance of ^15^N in the sample before (atom% ^15^N_natural_) and after labeling (atom% ^15^N_labeling_) were used to determine newly taken up ^15^N in the samples as APE (atom% excess):

$$\;APE{\;}^{15}N=atom{\%}^{15}N_{natural}-\;\;atom{\%}^{15}N_{labelling}$$

To determine the content of ^15^N (g g^−1^ dry wt) in the sample, we calculated:

Nsample15=APE15N100×Nsample

where Nsample represents the content of total N (g g−1 dry mass) in the sample.

The relative root uptake efficiency for ^15^N was determined as the ratio of the total amount of ^15^N in the roots present in the labeled soil volume and the total amount of ^15^N in the labeled soil (after Lang et al., 2017):

N15uptake efficiency (%)=(Nfine roots 15× fine root biomass+15Ncoarse roots ×  coarse root biomass)Nsoil15×soil weight×100

^15^N uptake efficiency was determined 4 h after label application.

^15^N recovery was determined as:

Recovery (%) =(Nfine roots15× fine root biomass+15Ncoarse roots× coarse root biomass+15Nsoil×soil weight)1.2×100

where 1.2 refers to the amount of 1.2 mg ^15^N applied to the soil.

### Data Analyses

Statistical analyses were performed using R 3.3.1 (R Development Core Team, 2008). When necessary to meet assumptions of normality and homoscedasticity of residuals, data were square-root- or logarithm-transformed. To test for significant effects of the land-use system on root biomass and morphology, soil characteristics, and plant N concentrations and uptake efficiency, one-way ANOVA was performed using “car” package (Allen and Shachar-Hill, 2009). The independent two-sample t-test *post hoc* Tukey test, implemented in the “multcomp” package (Hothorn et al., 2008), was used for multiple comparisons of treatment means. Repeated measures analysis of variance was conducted to determine whether the amount of ^15^N in the soil and roots varied with the land-use system and sampling time points. The mixed-effects model describing the repeated measures analysis was conducted in “nlme” package (Pinheiro et al., 2017). To take into account the differences between the sample locations in plots, the *sample name* was included as a random variable in the model. The temporal autocorrelation structure of order one (Zuur et al., 2010) was modeled within levels of the *sampling names*, using the auto-correlation functions (ACF) plot of residuals. In the tables and figures, data are shown as means ± standard errors (SEs). Differences were considered significant at P ≤ 0.05.

## Results

### Soil and Root Characteristics

In the oil palm plantations, the soil had a higher bulk density (P = 0.001) and lower water content than in forest plots (P < 0.001, **Table 2**). The fine root biomass and the number of root tips per soil volume were 3.5 and 2.0 times, respectively, higher in the oil plantations than in the forests (P < 0.001; **Table 2**). However, the root vitality and ratio of the number of vital root tips to fine root biomass were significantly lower in the oil palm plantations in comparison with forest plots (P = 0.001; **Table 1**). The coarse root biomass did not vary with the land-use system (P = 0.699).

**Table 2:** Soil and root characteristics in the lowland rainforest areas and oil palm plantations.

	Rainforest	Oil palm
Soil bulk density (g cm^−3^)	0.99 ± 0.02 a	1.17 ± 0.03 b
Soil water (%)	24.14 ± 0.51 b	16.80 ± 0.51 a
Fine root density (mg cm^−3^)	2.07 ± 0.16 a	7.41 ± 0.62 b
Coarse root density (mg cm^−3^)	3.74 ± 0.22 a	3.53 ± 0.25 a
Root tips density (number cm^−3^)	1.00 ± 0.08 a	2.18 ± 0.16 b
Root tip vitality (%)	84.96 ± 2.78 b	76.45 ± 1.01 a
Root tips/biomass (number mg^−1^)*	0.46 ± 0.04 b	0.25 ± 0.03 a

*Indicates the ratio between number of vital root tips and fine root biomass.

Data are means ± SE, N = 45.

*Indicates the ratio between number of vital root tips and fine root biomass.

Different lower-case letters indicate statistically significant differences between the two land-use systems.

### N Status and ^15^N Recovery in Soil and Root Biomass

**Figure 1:**
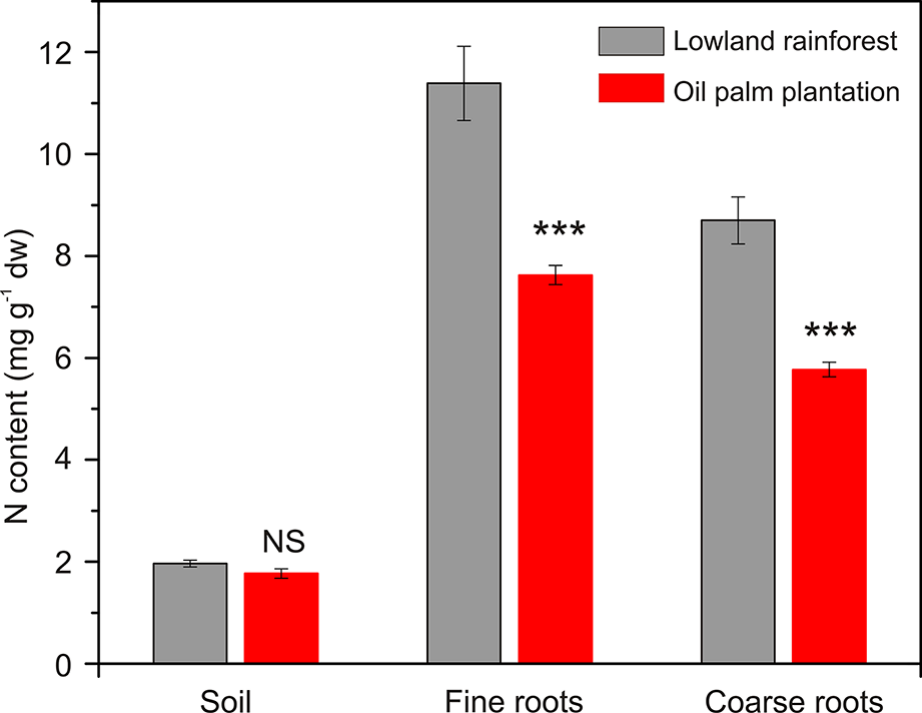
Nitrogen (N) concentrations in soil, fine roots, and coarse roots of oil palm plantations and rainforests. Data were measured in samples collected during a time course of 10 days. Values are means ± SE, n = 9. Land-use type effects were assessed by student t-test. ***P < 0.001, NS not significant.

Soil N contents were similar in the two land-use systems (P = 0.106), with values ranging from 1.5 to 2.0 mg N g^−1^ soil dry weight (**Figure 1**). The N contents both in fine and coarse roots were by one-third lower in the oil palm plantations than in the forests (P < 0.001, **Figure 1**).

**Figure 2:**
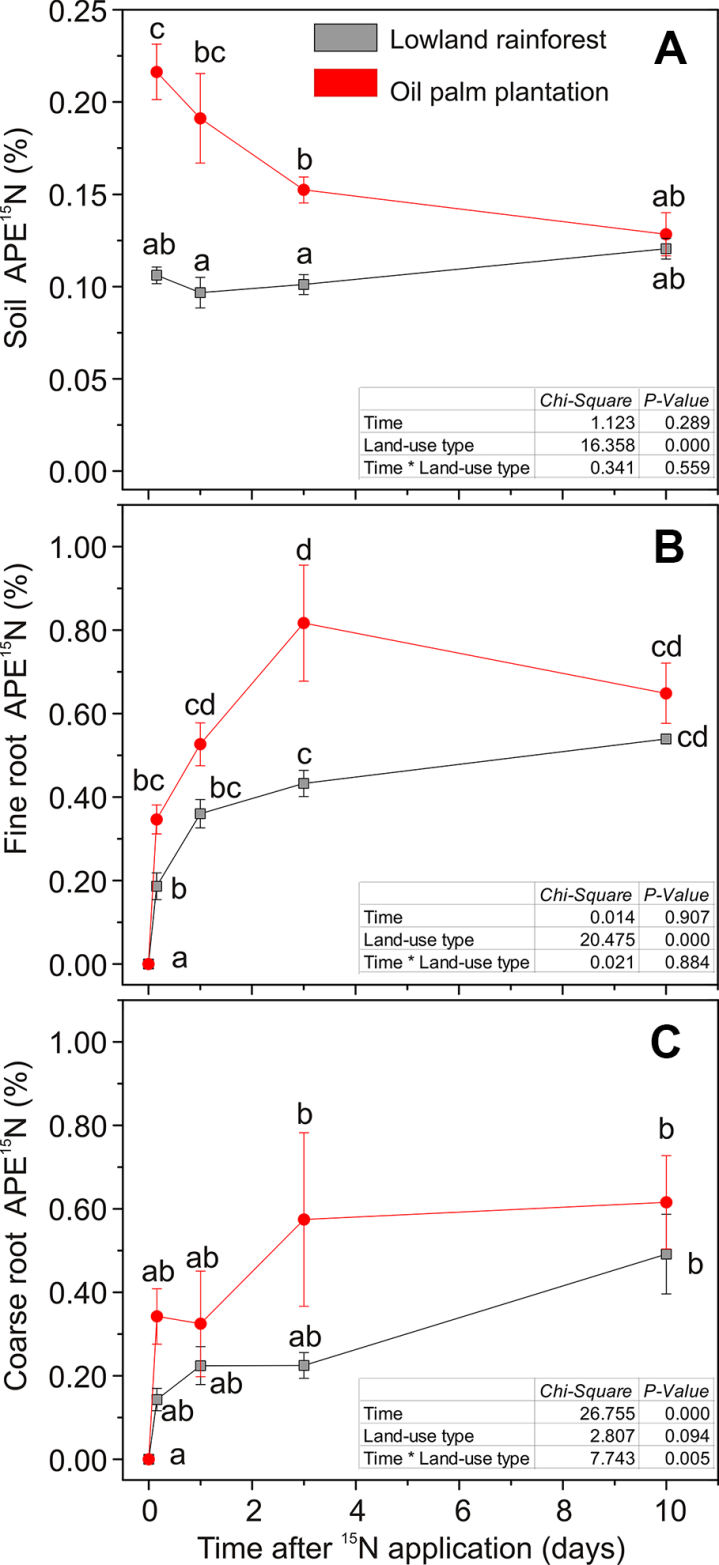
^15^N enrichment in: **(A)** soil; **(B)** fine roots; **(C)** coarse roots during a time course of 10 days after label application. At time point 0, soil was irrigated with 40 ml of 2 mM ^15^NH_4_Cl solution. Data show atom-% excess (APE) calculated as the difference of ^15^N after and before labeling for each compartment. Data are means ± SE, n = 9. Different letters indicate statistically significant differences, P < 0.05. Table inset: statistical results from repeated measures ANOVA.

**Figure 3:**
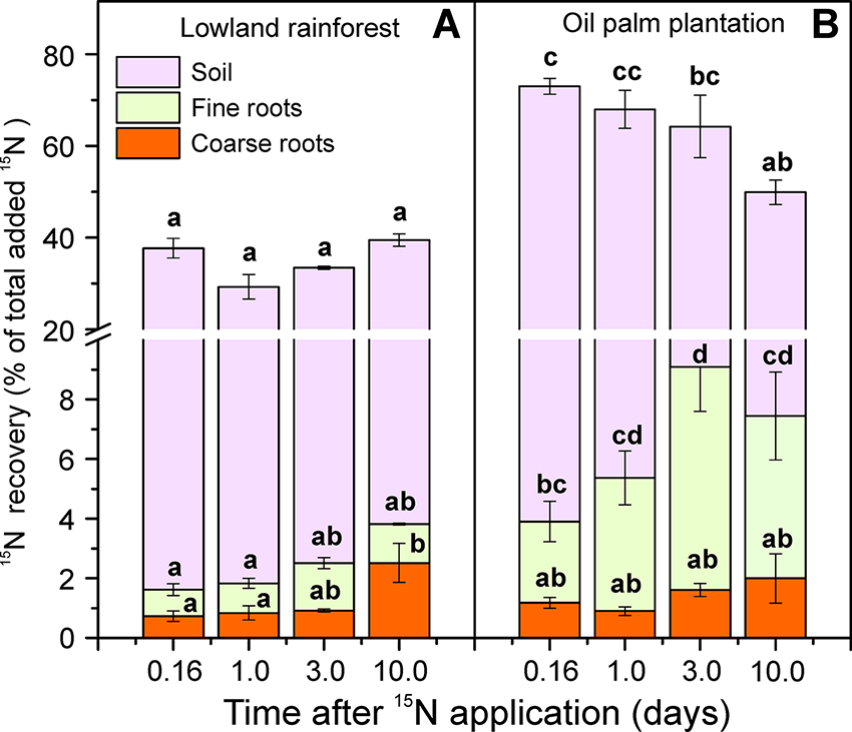
Recovery (%) of the applied ^15^N in soil cores (depth: 0.1 m, diameter: 0.05 m diameter) in the tropical lowland rainforests **(A)** and oil palm plantations **(B)**. The amount ^15^N that was present in each fraction (soil, fine roots, coarse roots) was determined separately, during a time course of 10 days. The applied amount (100%) of N corresponded to 1.2 mg ^15^N. Bars indicate means ± SE, n = 9. Different letters indicate significant differences between means at P < 0.05.

Label application resulted in differences in soil ^15^N enrichments between the two land-use systems (χ^2^ = 16.35, P < 0.001; **Figure 2A**). In the rainforests, soil APE^15^N was about 0.1% throughout the time course of 10 days after label application. In the oil palm plantations, APE^15^N was initially twice as high (0.2%) and declined to 0.1% 10 days after labeling (**Figure 2A**). Differences in the dynamics of soil ^15^N enrichment were also evident when considering ^15^N recovery. Four hours (0.16 day) after the label application, 70% of the applied ^15^N was recovered in the soil in the oil palm plantations, while in the forests 36% were retrieved (**Figure 3**). In all but the last (10.0 days) harvest after labeling, total^15^N recovery was higher in oil palm plantations than in forests (**Figure 3**). Ten days after labeling, ^15^N recovery was similar in both land-use systems because the recovery declined in oil palms and was stable in the forest.

Plant uptake of ^15^NH_4_^+^ occurred immediately after the label application in both land-use systems (**Figure 2B**). The mean value of recovered ^15^N in the roots across all harvests, following the labeling, was more than double in the oil palm plantations (6.4%) than in forests (2.4%, **Figure 3**). However, the fine roots in oil palm plantations and rainforests showed different kinetics of ^15^N uptake (χ^2^ = 20.47, P < 0.001; **Figure 2B**). In rainforest roots, a strong ^15^N enrichment was found within 1 day with a further slow increase within the next 10 days after labeling (**Figure 2B**). In the oil palm roots, the maximum ^15^N enrichment occurred 3 days after labeling, and afterwards, the ^15^N enrichment showed a declining trend (**Figure 2B**).

A significant ^15^N enrichment in the coarse roots was apparent 3 and 10 days after label application in oil palm plantations and in rainforests, respectively (**Figure 2C**). APE^15^N was not significantly different between the land-use type. The significant temporal increase in coarse roots (χ^2^ = 26.75, P < 0.001; **Figure 2C**) that was not observed in fine roots or soil indicates the transport of labeled N in the plant.

**Figure 4:**
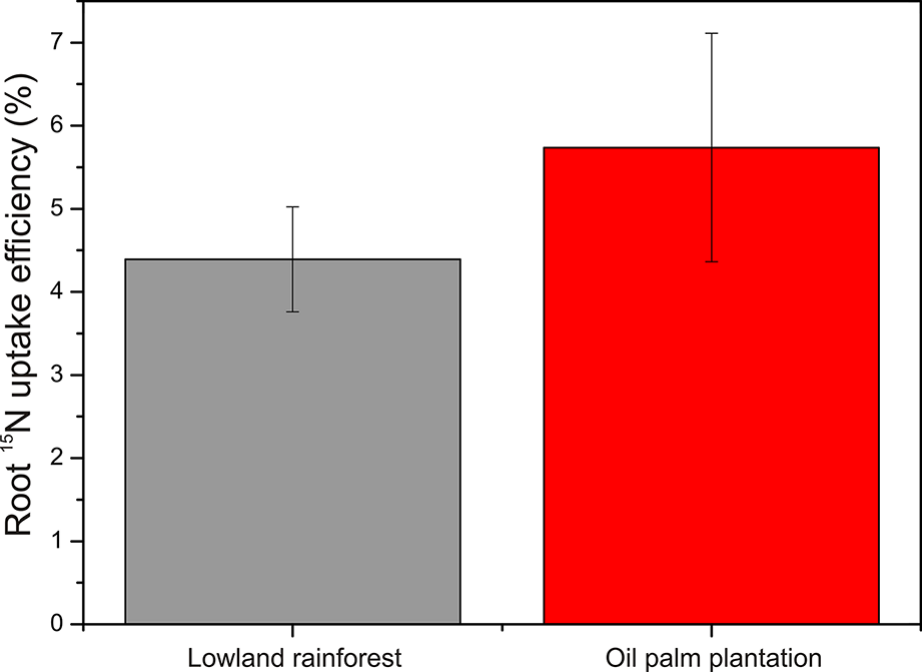
Root ^15^N uptake efficiency in the lowland rainforests and oil palm plantations determined 4 h after label application. Bars indicate means ± SE, n = 9.

The relative root ^15^N uptake efficiency showed no differences in ^15^N uptake efficiency between oil palm and rainforest roots (P = 0.975; **Figure 4**).

## Discussion

In this study, we measured how much from the soil-applied NH_4_^+^-^15^N was taken up by roots and calculated plant N uptake efficiency in a tropical lowland rainforest-oil palm plantation conversion systems. We found that total N contents were lower in oil palm than rainforest roots. We demonstrated in this study that the mechanism behind these findings is not a difference in root N uptake efficiency for NH_4_^+^-^15^N between oil palm and rainforest roots. This result is surprising since root vitality and number of root tips per root biomass were higher in the rainforests than oil palm plantations. Moreover, another study in the same experimental area reported more healthy roots and higher colonization rates by mycorrhizal fungi (Sahner et al., 2015), which are beneficial for N acquisition (Hodge et al., 2001; Hodge et al., 2010), in the forest than oil palm plantations. A possible explanation for our unexpected result might be that forest roots may endure a stronger microbial competition for N uptake than oil palm roots in managed soils. For example, a decrease in root competitiveness for N uptake at the advantage of soil microbes in the forest as compared with plantation has been reported in a Borneo lowland rainforest transformation system (Hamilton et al., 2016). Nitrogen immobilized by the microbial community may temporarily reduce soil N availability for the plant, but immobilized N can become again available in the rhizosphere due to microbial turnover (Groenigen et al., 2015). This mechanism may improve plant N nutrition under long term. It might be present in the rainforest, where plant-associated microbial communities are usually abundant, well-adapted, and stable (Brundrett and Tedersoo, 2018), in contrast to oil palms the soil community shifted from beneficial to pathogen fungi (Brinkmann et al., 2019). Another explanation for our findings is that, although the soil mineral N pool is dominated by NH_4_^+^ (Allen et al., 2015), in the mixed rainforests roots access various other N pools and minimalize N uptake from a sole source (i.e., NH_4_^+^-N) as compared with monocultures. This is because the high plant diversity can give rise to species complementarity in N resource-use in keeping with individual preferences and abilities to take up N (Ashton et al., 2010; Boudsocq et al., 2012; Richards et al., 2010; Wang and Macko, 2011). Plants vary in their strategies to acquire N from the soil (Weigelt et al., 2005). For example, there are species, which take up N as either NO_3_^−^, NH_4_^+^ or amino acids, access N sources at different soil depths (Boudsocq et al., 2012; Britto and Kronzucker, 2013; Wang and Macko, 2011), or are associated with N fixing prokaryotes and mycorrhizal fungi for N supply (Brundrett and Tedersoo, 2018).

Oil palms are highly productive species forming an extensive root system (Lamade et al., 1996; Nelson et al., 2014). Higher root biomass in oil palm plantations than in rainforests has often been reported (this study; Violita et al., 2016; Kotowska et al. 2016; Guillaume et al., 2018). Higher root density resulted in the enhanced recovery of the added ^15^N. However, the initial ^15^N recovery in our experimental soil volume was also higher in the plantation than in the forest. The reason was probably that the forest soil was less compact and had a higher water content than the plantation soil, leading thus initially to a broader spread of the label (Castellano et al., 2013). To accommodate for the differences in ^15^N availability in soil, we determined uptake efficiencies for ^15^N of oil palm and forest roots. Notably, no significant differences were detected. This result supports our alternative hypothesis, posed at the beginning of this study that at similar N uptake efficiency and higher biomass productivity, the N content of oil palm roots is lower than that of mixed forest roots. As a consequence, the ^15^N enrichment (APE) of the oil palm roots was higher than that of forest roots.

The ^15^N enrichment (APE) provides information on the dynamics of the label during the time course of the experiment. In the oil palm soil the ^15^N label declined, which could be due to plant uptake and fast N transformation by nitrification (Arnold et al., 2008; Koehler et al., 2009). The nitrification rate in our plots was 1.9 mg N kg^−1^ soil day^−1^ (Allen et al., 2015). Thus, the NH_4_^+^ applied here was probably nitrified in short time and subjected to losses through leaching or denitrification (Powlson, 1993; Arnold et al., 2008; Koehler et al., 2009). Based on different kinetics for ^15^N dynamics in oil palm and forest soil, we would have to postulate that N losses from the system were larger for oil palms than for rainforests. This suggestion is supported by Kurniawan et al., 2018. However, we cannot exclude that the stronger decline of ^15^N in the soil of oil palms was caused by larger absorption due to a larger root system and transport to aboveground sinks such as fruit bunches of the oil palms. The enrichment of ^15^N (APE) in coarse roots indicates N transfer within the plant and suggests that the transfer was faster in oil palms, where ^15^N enrichment occurred already 3 days after labeling than in forest trees, where a significant enhancement occurred at later time points. Our data support that both processes, soil turnover, and plant N uptake and translocation, affected soil N dynamics.

It is important to bear in mind that the experimental design of our study does not enable us to scale up from a tree physiology-based approach to higher areal levels because the variability of root biomass distribution in plantations and forests is high. For example, in oil palm plantations root biomass can vary by one order of magnitude around the trunk (Nelson et al., 2014) and up to 50% within the palm circle (Lamade et al., 1996). The greatest root biomass and highest physiological activities occur approximately 1 m from the trunk (Nelson et al., 2014), which is the distance where we collected our samples. We found in the oil palm plantation root masses in range similar to those reported by Lamade et al., (1996) but higher than those in some other studies (Kheong et al., 2010). To obtain representative estimations for the amounts of N taken up by oil palm roots at the plantation level, the management zones (palm circle, interrows, and front piles, cf., Darras et al., 2019) have to be included and the high variability of the oil palm root architecture must be considered. Future research should, therefore, seek to address these issues by enhancing the number of sampling points at different distances from the oil palm trunk and various depths in each of the management zones. Despite of these limitations, our study provided important insights into N physiology of oil palm and forest roots.

This work revealed that the lower root N contents, often found in roots of oil palms as compared with rainforest roots (this study, Sahner et al., 2015; Violita et al., 2016), are not based on lower efficiencies of oil palms to take up N from the soil. The differences are likely based on distinct plant-specific patterns of internal utilization of the absorbed N, highlighting a strong capacity of oil palms to acquire N. Thus, this study lends support to previous findings that the commonly applied high N fertilization rates may not be needed for maintaining the oil palm yields (Dubos et al., 2017; Darras et al., 2019). Collectively, these studies point towards opportunities to implement more sustainable management strategy involving a lower risk of N losses.

## Author’s Note

Research permit (Kartu Izin Peneliti Asing, permission number: 333/SIP/FRP/SM/IX/2012) was issued by the Ministry of Research and Technology RISTEK (Kementrian Ristek dan Teknologi, Jakarta, Indonesia). The Research Center for Biology of the Indonesian Institute of Science LIPI (Lembaga Ilmu Pengetahuan Indonesia, Jakarta, Indonesia) recommended issuing a sample collection permit (Rekomendasi Ijin Pengambilan dan Angkut (SAT-DN) Sampel Tanah dan Akar, number: 2696/IPH.1/KS:02/XI/2012). Collection permit (number: S.16/KKH-2/2013) and export permit (reference number: 48/KKH-5/TRP/2014) were issued by the Directorate General of Forest Protection and Nature Conservation PHKA (Perlindungan Hutan dan Konservasi Alam, Jakarta, Indonesia) under the Ministry of Forestry of the Republic of Indonesia. The Chamber of Agriculture of Lower Saxony (Plant Protection Office, Hannover, Germany) issued the import permits (Letter of Authority, numbers: DE-NI-12- 69 -2008-61-EC, DE-NI-14- 08 -2008-61-EC).

## Data Availability Statement

The raw data supporting the conclusions of this article will be made available by the authors, without undue reservation, to any qualified researcher.

## Author Contributions

Conceptualization: AP and RP. Field work: NE, UY, BI. Laboratory work: NE. Data analysis: AP and RP. Data curation: NE and RP. Writing—original draft preparation: RP. Writing—review and editing: AP. Funding acquisition: AP.

## Funding

This study was funded by the Deutsche Forschungsgemeinschaft (DFG, German Research Foundation) – project number 192626868 – SFB 990 (and the Ministry of Research, Technology and Higher Education (Ristekdikti)) in the framework of the collaborative German - Indonesian research project CRC990: Ecological and Socioeconomic Functions of Tropical Lowland Rainforest Transformation Systems (Sumatra, Indonesia) project B07, and Erasmus Mundus, EURASIA2 providing a scholarship to EN, and by CRC990 internal ABS research grants awarded to counterparts. Deutsche Forschungsgemeinschaft and Georg-August Universität Göttingen open access publication fund.

## Conflict of Interest

The authors declare that the research was conducted in the absence of any commercial or financial relationships that could be construed as a potential conflict of interest.

## Supplementary Material

Figure S1 Sampling sites in Harapan Province, Sumatra, Indonesia. Forestsites = HF, Oil palm planation sites = HO.
