# Supplementary figures and images for: Differences in Root Nitrogen Uptake Between Tropical Lowland Rainforests and Oil Palm Plantations
Source: Front Plant Sci. 2020 Feb 25;11:92. doi: 10.3389/fpls.2020.00092 (PMC7053111; doi:10.3389/fpls.2020.00092)

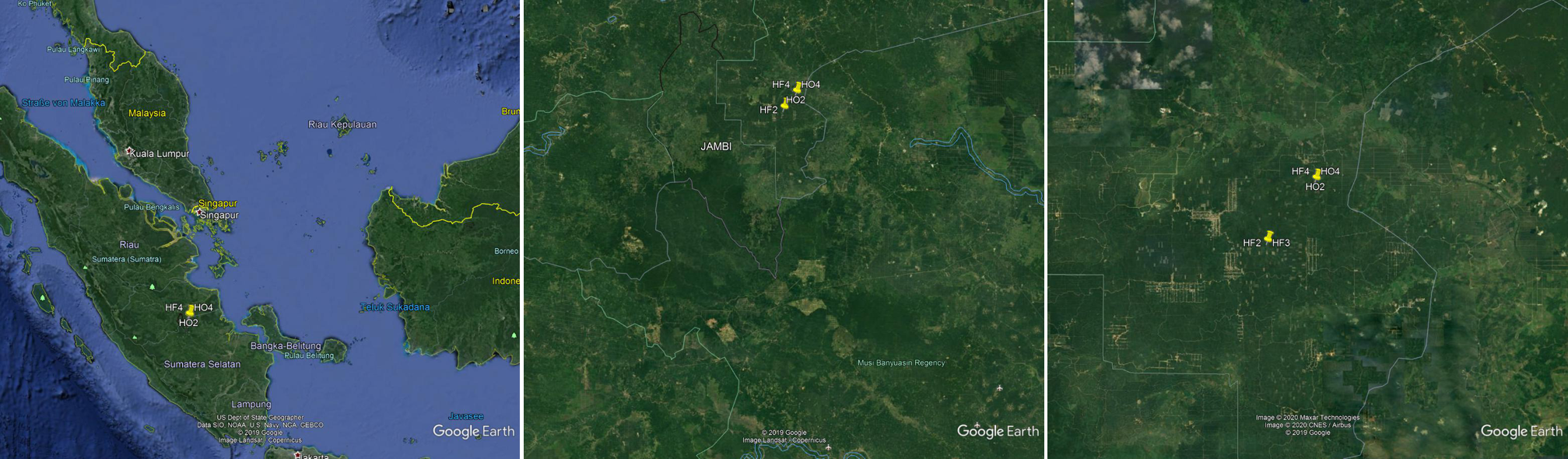

Supplement: Figure S1 — Sampling sites in Harapan Province, Sumatra, Indonesia. Forestsites = HF, Oil palm planation sites = HO. [file Image_1.tif]
